# A simple and rapid preparation of smooth muscle myosin 2 for the electron microscopic analysis

**DOI:** 10.1186/s42649-023-00094-5

**Published:** 2024-01-02

**Authors:** Anahita Vispi Bharda, Hyun Suk Jung

**Affiliations:** Department of Biochemistry, College of Natural Sciences, Kangwon National University, Chuncheon, Gangwon 24341 Republic of Korea

**Keywords:** Smooth muscle myosin 2, 10S myosin, Protein purification, Phalloidin, Transmission electron microscopy

## Abstract

There has been an increase in the demand for purified protein as a result of recent developments in the structural biology of myosin 2. Although promising, current practices in myosin purification are usually time-consuming and cumbersome. The reported increased actin to myosin ratio in smooth muscles adds to the complexity of the purification process. Present study outlines a streamlined approach to isolate smooth muscle myosin 2 molecules from actomyosin suspension of chicken gizzard tissues. The procedure entails treating actomyosin for a brief period with actin-binding peptide phalloidin, followed by co-sedimentation and short column size exclusion chromatography. Typical myosin molecule with heavy and light chains and approximately 95% purity was examined using gel electrophoresis. Negative staining electron microscopy and image processing showed intact 10S myosin 2 molecules, proving that phalloidin is effective at eliminating majority of actin in the form of F-actin without dramatic alteration in the structure of myosin. The entire purification discussed here can be completed in a few hours, and further analysis can be done the same day. Thus, by offering quick and fresh supplies of native myosin molecules suited for structural research, specially cryo-electron microscopy, this innovative approach can be adapted to get around the drawbacks of time-intensive myosin purifying processes.

**Supplementary Information:**

The online version contains supplementary material available at 10.1186/s42649-023-00094-5.

## Introduction

Myosin 2 is a conventional motor protein that performs mechanical work in cells with actin, utilizing chemical energy from ATP hydrolysis (Geeves and Holmes 1999). In terms of function, myosin 2 can exert powerful contractile forces in the muscles or participate in a wide range of processes like cell division, cell motility, neural development, and dysfunctional activities in the non-muscular system (Ma and Adelstein 2014; Shutova and Svitkina 2018; Vicente-Manzanares et al. 2009). A single myosin 2 molecule has a pair of homologous heavy chains and two pairs of light chains, the regulatory light chain (RLC) and the essential light chain (ELC). The amino terminal of the heavy chains forms the globular head which comes with a set of light chains while the carboxy terminal extends as α-helical coiled coil tail. The actin binding and ATPase activity of the globular heads are carried out by the motor domain. The tail however, commits in the regulation of myosin by folding back onto the head to generate a folded hairpin conformation (Burgess et al. 2007; Craig et al. 1983; Jung et al. 2008a, b). The conserved, shut-off state of the molecule that prevents ATPase activity is represented by the folded configuration of the molecule, also referred as the 10S myosin. The two bent heads interacting with one another and the triple segment tail folding make up the key structural elements of a typical 10S myosin monomer (Cross et al. 1986; Jung et al. 2008a, b; Wendt et al. 2001). The two conformers, 10S and 6S were ascribed on the basis of sedimentation coefficient of the monomer where the folded form is called 10S while the extended form is termed as the 6S monomer (Suzuki et al. 1982).

There is a pressing need to acquire purified myosin 2 as many studies recognize the structural, functional and regulatory relevance of its folded form in different diseases (Scarff et al. 2020; Yang et al. 2020). Purification of smooth muscle myosin 2 has been an age-old practice that comes with numerous strategies targeting myosin extraction from tissues. Given, the higher molecular ratio of actin to myosin in smooth muscles, it becomes necessary to eliminate actin and other accessory proteins to obtain single, homogenous preparation of myosin. Sources like gizzard tissue when homogenized in a suitable buffer can provide generous yield of smooth muscle myofibrils. Actin and myosin making up the major proteins of myofibril, can be separated by centrifugation under relaxing conditions (ATP and low Ca^2+^). However, persistent weak interaction cycle between actin and myosin interferes with the separation process. As a result, myosin would still contain significant amount of actin as well as other accessory proteins, necessitating further resuspension or overnight dialysis, clarification by ultracentrifugation, and chromatography. This explains the additional steps needed that increase the total amount of time for purification.

In this study, we target actin contamination using actin binding protein to eliminate the existing actin from the myosin suspension. Among various actin binding proteins, phalloidin has been studied extensively in F-actin stability. Phalloidin is a small (80 Da) cyclic peptide obtained from the death cap mushroom, *Amanita phalloides* (Lynen and Wieland 1938). By virtue of its capability, phalloidin binds to stabilize the F-actin in a stoichiometric fashion inversing the dissociation of F-actin (Estes et al. 1981; Wieland and Govindan 1974). When coupled with phalloidin, F-actin can withstand adverse conditions of high salt or ATP that would otherwise result in dissociation to monomeric G-actin (Dancker et al. 1975; Lengsfeld et al. 1974). F-actin tethered with phalloidin during centrifugation co-sediments as stable filaments that resist multiple rounds of association and dissociation. Assuming the minimal actin myosin interaction in the relaxing buffer, phalloidin treated F-actin will sediment out to leave behind a supernatant of myosin 10S molecules. This is true since the long strands of F-actin stabilized by phalloidin sediment readily than myosin molecules in the actomyosin mixture. Phalloidin helps to separate actin from myosin thus forming the basis of purification. SDS-PAGE analysis revealed that myosin purity following phalloidin treatment was significantly higher from the untreated control and a quick column chromatography procedure then increased the purity to over 95%. In addition, the classic folded structure of 10S myosin was observed by TEM without the presence of any major actin contamination. Consequently, myosin produced by this approach is physiologically and structurally viable for immediate application to electron microscopy as well as any structural studies as an advantageous scheme to quickly prepare myosin II molecules from gizzard tissues.

## Materials and methods

### Extraction of actomyosin fibrils

**Fig. 1:**
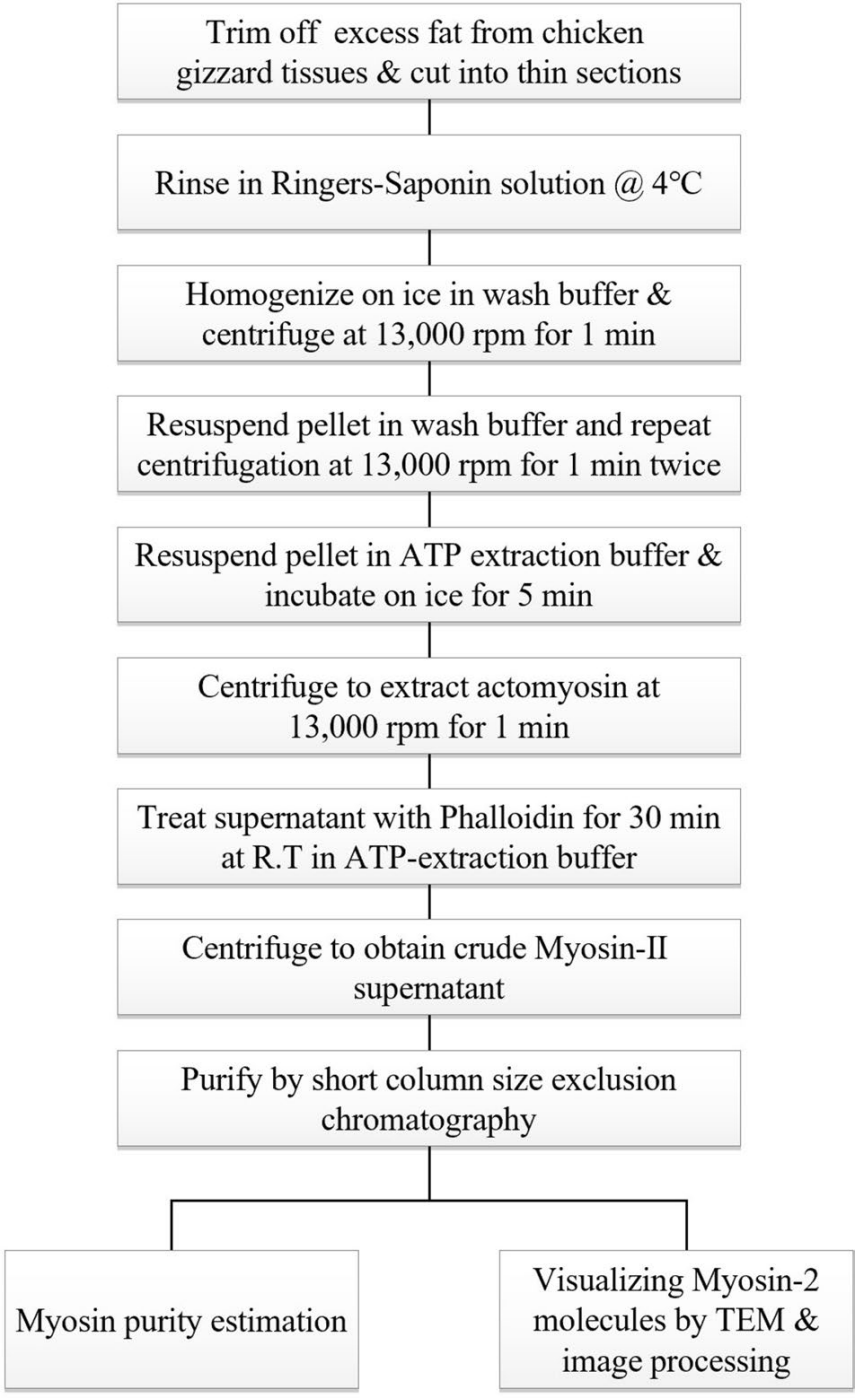
Workflow of the preparation and analysis of Myosin-II from chicken gizzard tissues using phalloidin. Details of the buffers and other conditions have been described in the “Materials and methods” section

Smooth muscle myosin II was purified from chicken gizzard tissue by modifying the methods of (Persechini and Hartshorne 1983) and (Burgess 2007). Fresh chicken gizzards were obtained from a local poultry house, washed in Ringers solution and rapidly eliminated of the fat tissues. The cleaned muscles were weighed (2 grams) and cut into small sections in wash buffer (150 mM KCl, 2 mM EGTA, 2 mM MgCl_2_, 0.5 mM DTT, 10 mM MOPS, pH 7.5) with 0.1% Saponin and stirred for a few hours at 4°C. This washing is optional but it helps soften tissues for better homogenization. Rinsed tissues were suspended in 3 volume (with respect to original muscle weight) fresh washing buffer without Saponin, minced finely and homogenized using Polytron homogenizer (WiseTis-HG-15D, Daihan Co., Wonju, South Korea) for 10 sec on ice followed by centrifugation at 13,000 rpm for 1 min. The resulting supernatant was discarded and the pellet was re-suspended in fresh washing buffer and centrifuged again. A total of two or maximum three wash cycles was performed till the supernatant turned transparent. In order to extract actomyosin, the pellet was suspended in 2 volume extraction buffer (150 mM KCl, 2 mM EGTA, 2 mM EDTA, 0.5 mM DTT, 5 mM ATP, 10 mM MOPS, pH 7.5), incubated on ice for 5 min and centrifuged at 13,000 rpm for 1 min to collect the final actomyosin supernatant. The major steps of the purification protocol have been summarized briefly in (Fig. 1).

### Separation of actin by phalloidin treatment under low-speed centrifugation

**Fig. 2:**
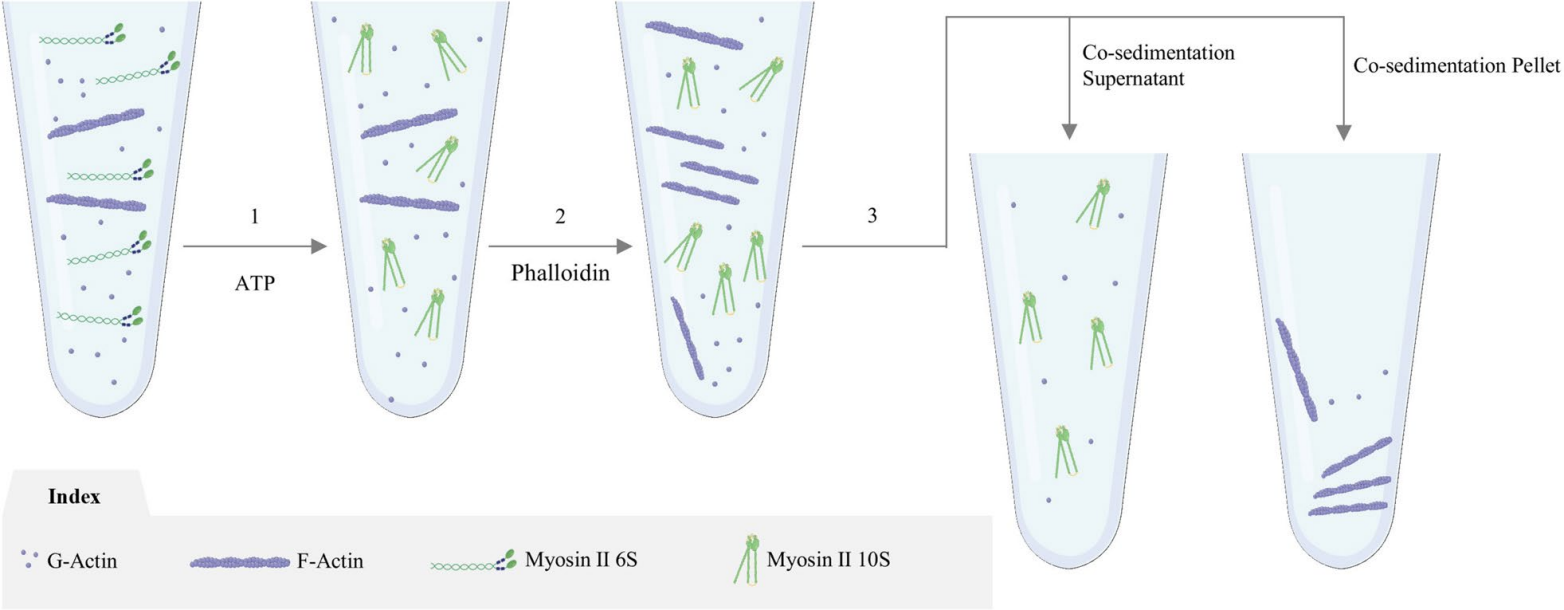
Graphical illustration of the myosin-II purification using phalloidin. Conceptual interpretation of the reactions undertaking at the molecular level during the course of purification. Every step is a consequence of the treatment or condition applied in the previous step

The actomyosin supernatant (~1 ml) was equilibrated at R.T to which 50 μl (1 mg/ml in extraction buffer) phalloidin (Sigma P2141) and allowed to react for 30 min (it is recommended to pre determine the approximate ratio of actin from actomyosin solution before phalloidin treatment for best results). Following the addition of 1 mM ATP, the solution was centrifuged at 15,000 rpm (~22,000 x g) for 1 hour and the crude myosin supernatant with reduced actin contamination was ready to be purified by short column chromatography. Principle underlying the phalloidin reaction to actomyosin solution is depicted in (Fig. 2).

### Short column chromatography

Sepharose 4B resin filled column was prepared using a small glass Pasteur pipette. With the assistance of a long toothpick, a small piece of filter cloth folded into two halves was plugged at the bottom of the pipette. In order to prevent any bubbles from forming in the column, resin was injected drop by drop and a small pressure was created using a rubber stopper from the top. This was allowed until 70% of the column was filled with the resin. The column was ready to be used and given at least 2 column washes with 20% ethanol and distilled water each before being equilibrated with 3 column volume short column buffer i.e., same as the wash buffer previously indicated. Equilibration buffer with additional 0.5 mM ATP was run through the column before the crude myosin solution was applied. The fractions were then retrieved for analysis and used the same day.

### SDS PAGE gel analysis

SDS-polyacrylamide gel electrophoresis was carried out as per (Laemmli 1970) in 4-20% gradient separating gel and a 4% stacking gel with 1 mm thickness and electrophoresed at 200 V for 50 min. The resulting gel was stained with freshly prepared Coomassie brilliant blue R-250 and allowed to gradually de-stain overnight in 40% methanol and 10% acetic acid solution. The density of each band was analyzed using the ImageJ tool to estimate the purity of the myosin in the solution.

### Transmission electron microscopy & image processing

**Fig. 3:**
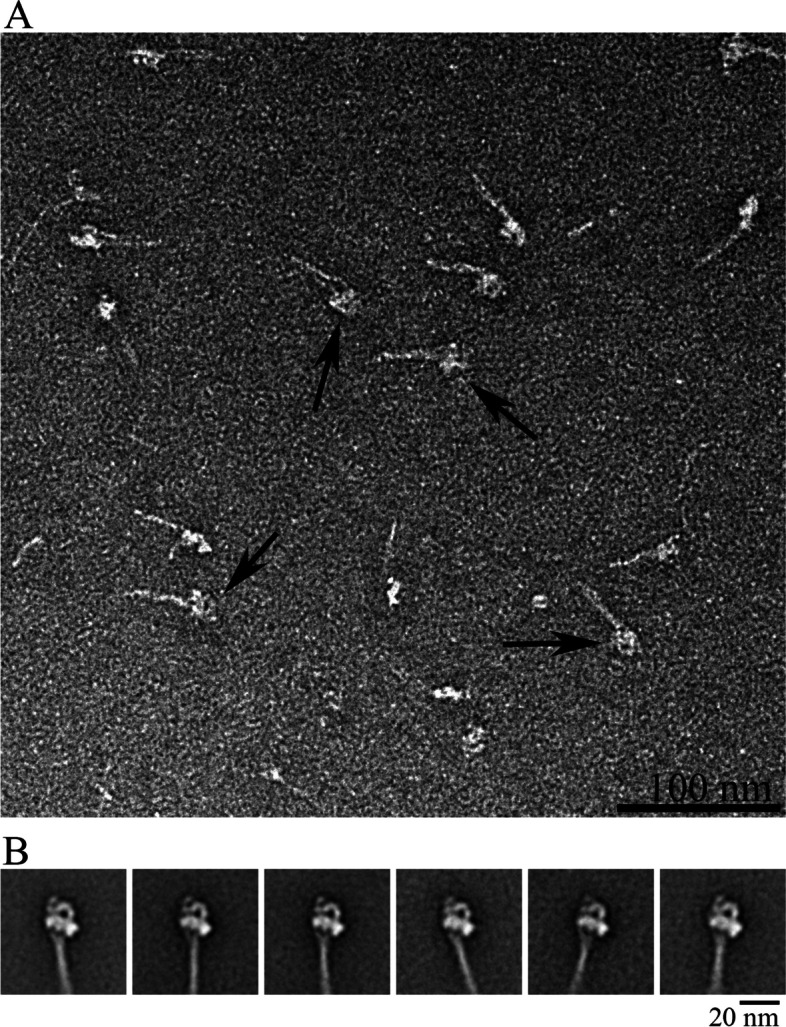
TEM analysis of myosin 10S molecules. **a** Myosin obtained from the supernatant post phalloidin treatment was diluted with ATP, MgCl_2_ low salt buffer and cross-linked with 0.1% glutaraldehyde to a final concentration of 100 nM. Black arrows show the head region of the folded myosin molecule. Scale bar, 100 nm. **b** Alignment and classification of the particles obtained from micrographs was done with 2500 compact particles using SPIDER. The head aligned images of the class averages show the general variance in the orientation of the head and tail. Scale bar, 20 nm

The purified myosin fraction was diluted with wash buffer containing ATP (150 mM KCl, 2 mM EGTA, 2 mM MgCl_2_, 0.5 mM DTT, 0.5 mM ATP, 10 mM MOPS, pH 7.5) to give a final concentration between 50-100 nM. This protein solution was cross-linked for 1 minute with 0.1% glutaraldehyde at R.T. (Jung et al. 2011). 5 μl of this mixture was applied to a pre glow-discharged (Harrick Plasma, NY) carbon-coated grid followed by negative staining using 1% uranyl acetate. The grid was allowed to air dry and examined under a transmission electron microscope (Tecnai T10, FEI, USA) operated at 100kV. Images were captured on a US1000 CCD camera (Gatan, USA) at a magnification of 34,000 (0.32 nm/pixel). Instrumentation was used at Kangwon Centre for Systems Imaging. The micrographs (Fig. 3) were used to extract myosin molecules for single particle analysis using SPIDER. Negative stained field of myosin molecules and class averages of single myosin particles were adopted with an appropriate scale bar using Adobe Photoshop.

## Results

Our primary objective was to obtain intact myosin molecules in the fewest possible steps and least contamination. We placed more emphasis on the sedimentation of actin than myosin to accomplish this. F-actin depolymerization was prevented by treating actin from actomyosin mixture with phalloidin. Preventing any potential harm to myosin during the process, phalloidin was dissolved in extraction buffer (see materials and methods) as opposed to its typical solvent, methanol. The actin polymerizing capability of phalloidin in the same buffer was tested by using skeletal G-actin as a control. The reaction took place for 30 min at R.T followed by co-sedimentation to analyze the band pattern of actin. SDS PAGE with TEM analysis ensured active polymerization of actin by phalloidin under our buffer of choice (Fig. S1).

### Treatment of actomyosin with phalloidin

**Fig. 4:**
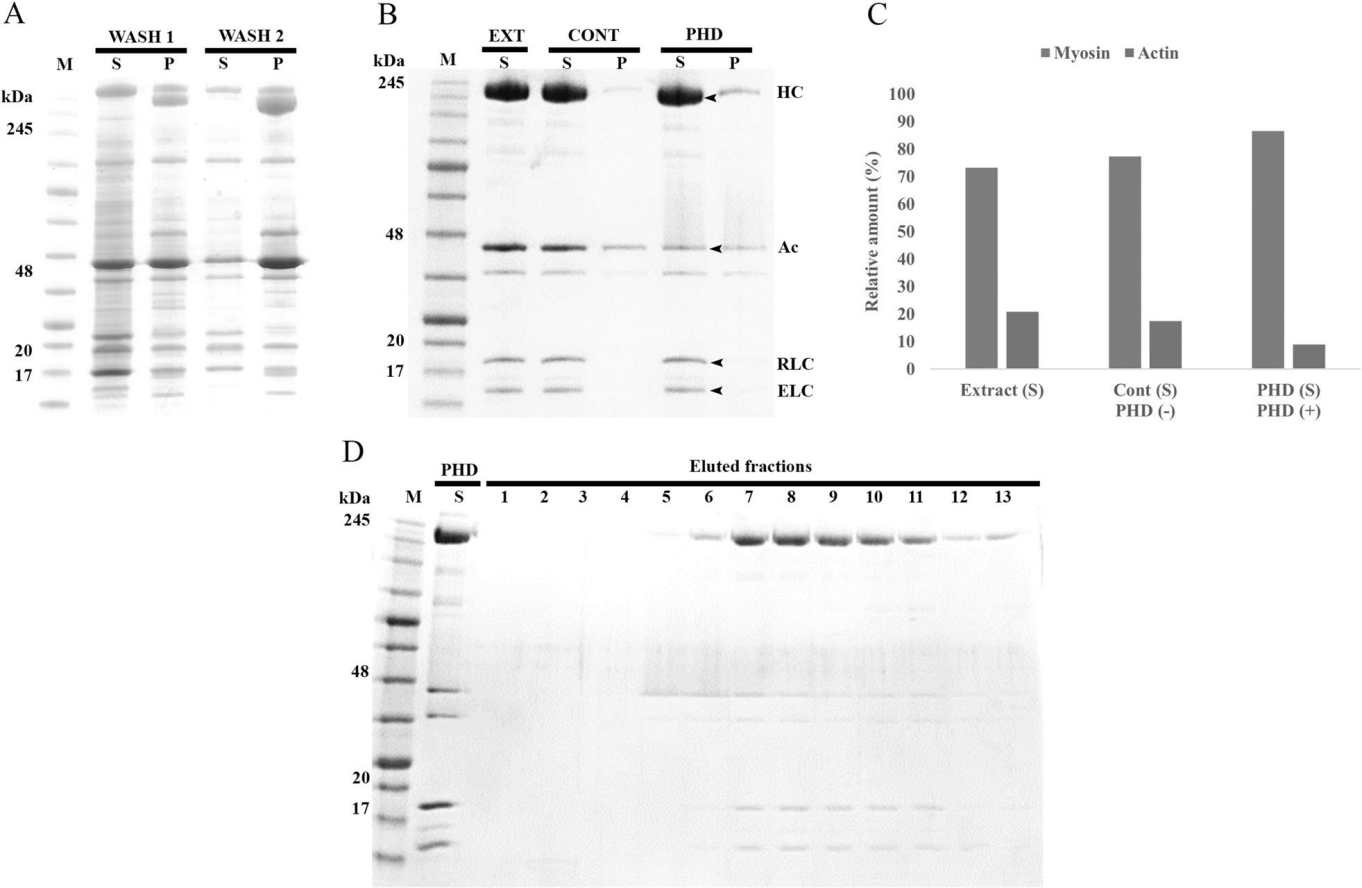
Step-wise SDS-PAGE and densitometric analysis of smooth muscle myosin-II purification. The proteins were electrophoresed on a 4-20 % gradient gel stained by Coomasie brilliant blue. (S) and (P) demonstrate supernatant and pellet, respectively collected during the washing **a**, extraction and co-sedimentation with or without phalloidin **b** steps of the purification after centrifugation. **c** Comparative densitometric analysis myosin and actin contents between the supernatants of the original actomyosin extract and with (PHD), or without (CONT) phalloidin treatment. **d** SDS-PAGE profile of eluted fractions from myosin purification using short size exclusion chromatography. S indicates the phalloidin treated supernatant; lanes 1-13 represent eluted fractions. Note that the bands representing Myosin Heavy Chains (HC), Regulatory Light chains (RLC) and Essential Light chains (ELC) and Actin (Ac) have been indicated by arrowheads respectively. The pre-stained protein markers were represented as (M). Note (EXT) designates actomyosin original extract

The initial washing steps during purification were intended on removing traces of saponin and auxiliary muscle proteins. As expected, SDS PAGE profile of the washing steps exhibited the characteristic gizzard fragments, with the thick and thin filaments present in the pellet (Fig. 4a). Pellet from the last washing step was treated with ATP under relaxing buffer condition (Fig. 4b) to extract myofibrils, leaving behind any inactive or strongly associated actomyosin. For phalloidin reaction, concentration of the actin from the actomyosin mixture was estimated by densitometric analysis of the gel bands. Based on this, an equimolar concentration of the phalloidin was calculated and added in the extracted myofibril mixture. This eliminates the possibility of unbound or free phalloidin being left in the final myosin solution. The time of reaction was kept to 30 min at R.T. to enable interaction with F-actin.

### Myosin purification post phalloidin treatment

As mentioned in introduction, we considered pelleting F-actin as a favorable means to retain myosin in the supernatant. Therefore, phalloidin was used to secure most of the actin population in its polymerized F-actin state that could be easily pelleted out upon sedimentation (Fig. 4b). Technically, F-actin being relatively longer and heavier than the folded myosin molecules, can be easily pelleted down under centrifugation. Although, lower speed centrifuges can’t help in complete F-actin pelleting. The phalloidin treated preparation showed lower percentage of actin than the untreated one, inclusive of the characteristic myosin heavy (200 kDa) and light chains (20 kDa and 17 kDa). On the contrary, control sample showed more actin enriched bands as a matter of that actin was still undergoing cycles of polymerization and de-polymerization and a possibility of ongoing reactions with myosin. The relative difference between the two preparations and purity of the final myosin obtained was determined by ImageJ analysis (bands representing the heavy chains, essential and regulatory light chains) (Fig. 4c). Here, the control showed no significant difference with the original actomyosin extract while actin content reduced over two-fold when reacted with phalloidin. The faint protein bands (below myosin Heavy Chains), are usually present in small quantities and were be removed by size exclusion chromatography. SDS Page analysis of the fractions obtained after chromatographic separation (Fig. 4d), showed the main myosin fractions with total actin contamination less than 5% by ImageJ analysis, leaving behind only the myosin, inclusive of heavy and light chains. Additionally, chromatographic purification ensured elimination of the residual muscle protein impurities and free phalloidin. Thus, the myosin obtained at this stage had over 95% purity and in our case, the yield produced to as much as 3 mg/ml of myosin.

### Electron microscopic analysis of the purified myosin preparation

Phalloidin treated myosin solution was subjected to negative staining electron microscopy to identify any alteration in its native structure. This would guarantee the efficacy of the purification method since TEM analysis can discern the structural integrity of protein. The final diluted myosin preparation was cross-linked with 0.1% glutaraldehyde before negatively staining with 1% uranyl acetate to stabilize the folded myosin molecules. While the control (untreated) supernatant showed many myosin 10S molecules, they came with the actin (Fig. S2, left panel). Treatment with phalloidin showed the typical folded 10S myosin molecules with rare appearances of actin in the supernatant (Fig. 3a). Instead, most of the actin was observed as long strands of F-actin entwined together in the pellet obtained after phalloidin treatment (Fig. S2, right panel). With the molecular size as small as 80 Da, it was not possible to visualize phalloidin nor were there any major changes in the structure of the 10S myosin molecules when the individual 10S myosin molecules were collected and subjected to 2D averaging (Fig. 3). This strongly supports our study that phalloidin did not impact the native, folded conformer of the 10S myosin molecules but selectively removed the actin contamination.

## Discussion

Tissues can be an excellent source to produce high yields of native proteins suitable for a wide range of biochemical and structural research (Hidalgo et al. 2001). Likewise, smooth muscle myosin can be excised readily from gizzard tissues. We came up with a simple, rapid and efficient approach to synthesize myosin molecules that are structurally stable and pertinent to conduct different myosin experiments. Our purification method makes the best use of the actin binding property of phalloidin to easily separate actin, a major contamination factor in myosin purification. It is well established that calcium (Ca^2+^) regulates smooth muscle contraction causes phosphorylation of the 20 kDa myosin light chains by myosin light chain kinase (MLCK) (Adelstein and Sellers 1987). Therefore, a Ca^2+^ independent environment will limit actin myosin cross-bridging. Despite, there are many factors that can sustain actomyosin association including the basal actin myosin kinetic cycles (Brenner et al. 1984). Targeting those factors that favor separation, we tried changing the buffer composition like the concentration of salt, ATP, cationic chelators, MgCl_2_ one by one. We also changed the pH of the buffer adapting to the most physiological state but failed to separate actin completely from myosin. In fact, we could sediment myosin to some degree but the actin would still remain suspended in the supernatant. After numerous unsuccessful attempts, we decided to use an actin-binding protein that helps pellet the actin to make sure myosin was still in the supernatant. Actin having smaller sedimentation coefficient than myosin would settle only if polymerized to form long strands of the filament. Phalloidin is one such compound that can stabilize actin against depolymerization amidst severe conditions (Dancker 1975). In our study, we skipped the customary overnight actin-phalloidin reaction with concerns regarding the stability of myosin. As an alternative, we added phalloidin after actomyosin extraction, regardless of whether the buffer conditions were favorable or unfavorable for actin polymerization (Bubb et al. 1994). The reaction proceeded for a limited time at R.T. ensuring that the stability of the proteins was not affected and simultaneously was adequate enough to removal actin (Fig. 4b). Even though the exact location of phalloidin-actin binding is not discovered, many studies have crucially pointed out that phalloidin binds at a region spanning the three subunits of actin from both the strands through intra and inter strand hydrophobic interactions (Mentes et al. 2018). This helped in stabilizing the F-actin, preventing depolymerization under any non-polymerizing condition as phalloidin critically delays the release of P_i_ (Barden et al. 1987; Dancker and Hess 1990; Pospich et al. 2020).

Assuming that F-actin can freely interact with myosin, we employed a relaxing condition (ATP, low salt and no Ca^2+^) without MgCl_2_ in the main extraction step, ruling out any possibility of actomyosin reconciliation. Actin that precipitated out was removed as a result, while myosin molecules were left behind in the supernatant (Fig. 4). However, myosin obtained at this stage still contained actin that could not be removed easily. As a result, phalloidin treatment became a favorable option to lend additional help in pelleting out the remaining actin via filament polymerization and sedimentation. Phalloidin-stabilized F-actin can withstand depolymerization and pellet by centrifugation. The final round of size exclusion chromatography eliminates the residual actin and other impurities. The entire purification did not last over few hours and myosin as the final product was retained in the supernatant itself. This approach focuses on improvising the quality of myosin molecules for structural analysis. The intention of letting myosin remain in the supernatant comes from the idea of protection against potential risks to the native myosin structure resulting from processes like ammonium sulfate precipitation. Through TEM, it was possible to show that the actin contamination, especially the monomers or short F-actin fragments were negligible when treated with phalloidin. In addition, image processing analysis demonstrated no significant modifications in the structural orientation of the myosin molecules under phalloidin treatment (Fig. 3a and b). Even with the optional step of muscle rinsing, the entire procedure takes less than six hours delivering super quick production of structurally stable myosin molecules.

## Conclusion

In conclusion, the results reported in this paper suggest that phalloidin in stoichiometric concentration can be beneficial in targeted actin removal during the purification of myosin molecules without major structural discrepancies. This approach can be useful in acquiring fresh myosin molecules directly from the crude actomyosin of gizzard tissues without the need to use old, stored vials of proteins. Significant advantages of this method lie in the simplicity of the equipment used and the minimal manual labor or equipment required. Such approach can be easily scaled up to produce myosin molecules assigned to high-resolution structural studies such as that of cryo-electron microscopy.

### Supplementary Information

**Additional file 1:** **Fig. S1.** SDS-PAGE and TEM analysis of skeletal actin. (a) Skeletal actin was used to confirm phalloidin activity on skeletal actin with respect to untreated control using gel electrophoresis. (A) indicates skeletal actin alone whereas (S) and (P) denote supernatant and pellet of the untreated, control skeletal actin (CONT) and phalloidin treated skeletal actin (PHD) respectively. (M) represents the pre-stained molecular weight protein marker. As expected, the actin was co-sedimented as long filaments when reacted with phalloidin which was not observed for the pellet of the control skeletal actin. (b) Negatively stained images of the untreated, control supernatant and (c) phalloidin-treated pellet of the skeletal actin. Scale bar, 100 nm.

** Additional file 2:** **Fig. S2.** Negatively stained field of the control untreated supernatant and phalloidin treated pellet fractions. (Left) The supernatant without phalloidin treatment, after centrifugation was collected, diluted and cross-linked using 0.1% glutaraldehyde to be observed under Transmission electron microscopy to check the presence of actin filaments. (Right) The pellet obtained after phalloidin reaction was diluted and cross-linked with 0.1% glutaraldehyde and analyzed by TEM for actin filaments. Scale bar, 100 nm.

## Data Availability

Not applicable

## Abbreviations

ATP: Adenosine triphosphate
Da: Dalton
KDa: Kilodalton
F-actin: Filamentous actin
G-actin: Globular actin
SDS-PAGE: Sodium dodecyl-sulfate polyacrylamide gel electrophoresis
TEM: Transmission electron microscopy
EGTA: Ethylene glycol-bis(β-aminoethyl ether)-N,N,N′,N′-tetraacetic acid
EDTA: Ethylenediaminetetraacetic Acid
DTT: Dithiothreitol
MOPS: 3-(N-morpholino)propanesulfonic acid
R.T.: Room temperature
kV: kilovolt
CCD: charge-coupled device
