# Supplementary figures and images for: A simple and rapid preparation of smooth muscle myosin 2 for the electron microscopic analysis
Source: Appl Microsc. 2024 Jan 2;54:1. doi: 10.1186/s42649-023-00094-5 (PMC10761634; doi:10.1186/s42649-023-00094-5)

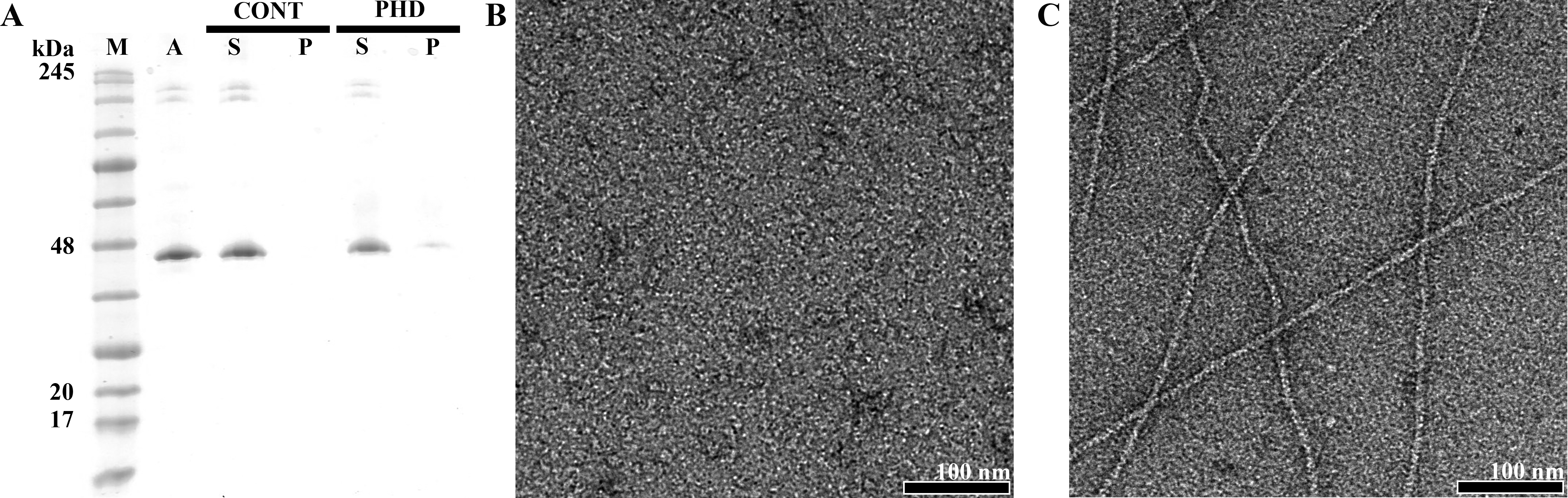

Supplement: Supplementary file 1 — Additional file 1: Fig. S1. SDS-PAGE and TEM analysis of skeletal actin. (a) Skeletal actin was used to confirm phalloidin activity on skeletal actin with respect to untreated control using gel electrophoresis. (A) indicates skeletal actin alone whereas (S) and (P) denote supernatant and pellet of the untreated, control skeletal actin (CONT) and phalloidin treated skeletal actin (PHD) respectively. (M) represents the pre-stained molecular weight protein marker. As expected, the actin was co-sedimented as long filaments when reacted with phalloidin which was not observed for the pellet of the control skeletal actin. (b) Negatively stained images of the untreated, control supernatant and (c) phalloidin-treated pellet of the skeletal actin. Scale bar, 100 nm. [file 42649_2023_94_MOESM1_ESM.tif]

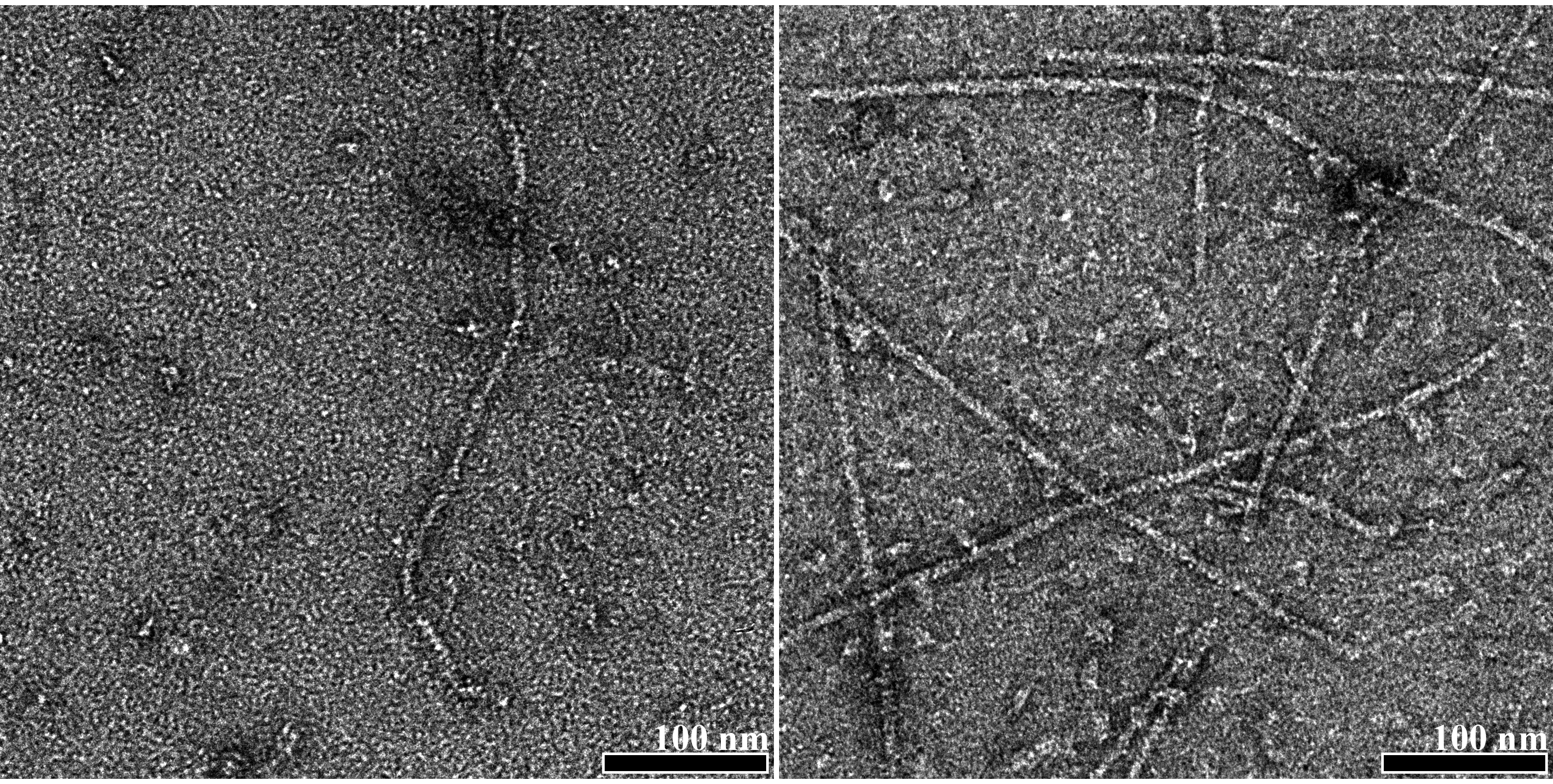

Supplement: Supplementary file 2 — Additional file 2: Fig. S2. Negatively stained field of the control untreated supernatant and phalloidin treated pellet fractions. (Left) The supernatant without phalloidin treatment, after centrifugation was collected, diluted and cross-linked using 0.1% glutaraldehyde to be observed under Transmission electron microscopy to check the presence of actin filaments. (Right) The pellet obtained after phalloidin reaction was diluted and cross-linked with 0.1% glutaraldehyde and analyzed by TEM for actin filaments. Scale bar, 100 nm. [file 42649_2023_94_MOESM2_ESM.tif]
